# Explainable machine learning for early detection of *Escherichia coli* urinary tract infections: integrating SHAP interpretation and bacterial epidemiology

**DOI:** 10.3389/fcimb.2026.1740707

**Published:** 2026-02-13

**Authors:** Jie Zhang, Ying-Ying Jiang, Ying Zhu, Chu-Ying Pan, Ling-Hui Yao, Ying-Ying Zheng, Shi-Yan Zhang, Jinbao Shi

**Affiliations:** 1 Department of Clinical Laboratory, Fuding Hospital, Fujian University of Traditional Chinese Medicine, Fuding, Fujian, China; 2 Department of Nephrology, Fuding Hospital, Fujian University of Traditional Chinese Medicine, Fuding, Fujian, China; 3 Department of Nephrology, Ningde Hospital of Traditional Chinese Medicine, Ningde, Fujian, China

**Keywords:** biomarkers, *Escherichia coli*, machine learning, Random Forest, SHAP, urinary tract infection, urine culture

## Abstract

**Background:**

*Escherichia coli* is the predominant uropathogen in urinary tract infections (UTIs), but culture-based identification is time-consuming. This study aimed to develop an explainable, culture-independent model to distinguish *E. coli* from other uropathogens using routinely collected clinical data.

**Methods:**

We retrospectively analyzed 308 hospitalized patients with culture-confirmed UTIs at Fuding Hospital, Fujian University of Traditional Chinese Medicine (January–December 2023), classified as *E. coli* (n = 158) or non–*E. col*i (n = 150). Species identification was performed using an automated microbiology system. Nineteen predictors (sex, urinary leukocyte grade, and 17 routine laboratory variables) were used. Associations with *E. coli* UTI were examined using univariate and multivariable logistic regression. A Random Forest (RF) classifier was developed with SHapley Additive exPlanations (SHAP) for interpretability. Data were split using a stratified 70/30 train–test split; 5-fold stratified cross-validation within the training set was used for hyperparameter tuning, and final performance (discrimination and calibration) was reported on the held-out test set. RF was additionally benchmarked against regularized logistic regression, calibrated linear SVM, and gradient boosting using the same protocol.

**Results:**

*E. coli* accounted for 51.3% of isolates, followed by *Enterococcus* spp. (18.5%) and *Klebsiella* spp. (7.8%). Compared with non–*E. coli* cases, *E. coli* infections were more common in females and showed higher lymphocyte counts (LYM), alanine aminotransferase (ALT), and albumin (ALB) (all P < 0.05). Multivariable logistic regression identified sex, LYM, and urinary leukocyte grade as independent predictors. On the held-out test set, RF achieved moderate discrimination (ROC-AUC = 0.66; average precision = 0.66) with calibration assessed by Brier score and calibration slope. SHAP highlighted Sex, LYM, and ALT as the most influential predictors and revealed patient-level heterogeneity in feature effects.

**Conclusions:**

*E. coli* remains the predominant pathogen among hospitalized UTIs. An explainable RF model using routine laboratory variables provided moderate, reproducible discrimination of *E. coli* vs non–*E. coli* UTIs and may support earlier decision-making while awaiting culture results.

## Introduction

Urinary tract infections (UTIs) are among the most prevalent bacterial infections across all age groups, with particularly high incidence in women and older adults (Mancuso et al., 2023). Between 1990 and 2019, the global number of UTI cases increased substantially, with cases rising from approximately 252 million to 405 million (Yang et al., 2022). *Escherichia coli* remains a leading uropathogen, accounting for a large proportion of uncomplicated community-acquired UTIs and remaining among the most common pathogens in healthcare-associated infections (Zagaglia et al., 2022; Chowdhury et al., 2024). Although empirical antibiotic therapy is often effective, timely and accurate pathogen identification is essential to guide targeted treatment, reduce antimicrobial misuse, and prevent serious complications such as pyelonephritis and urosepsis (Chardavoyne and Kasmire, 2020).

Standard diagnostic approaches, notably urine culture combined with biochemical identification methods, are widely regarded as the gold standard for confirming UTIs (Nelson et al., 2024). However, the turnaround time is typically 24–48 hours for organism identification and may extend to 48–72 hours when susceptibility results are included, which can delay clinical decision-making and increase reliance on broad-spectrum empirical antibiotics. Accordingly, diagnostic stewardship strategies have emphasized earlier risk stratification and targeted testing to optimize antimicrobial use (Morado and Wong, 2022).

In this context, machine learning (ML) techniques have garnered increasing interest for enhancing diagnostic precision by leveraging structured clinical data (Jeng et al., 2022; Shen et al., 2024). To support early, culture-independent differentiation of *E. coli* from non–*E. coli* UTIs using routinely available laboratory variables, we selected the Random Forest (RF) algorithm as a pragmatic, widely used baseline model because it can capture nonlinear relationships and higher-order feature interactions that are common in clinical laboratory data without requiring prespecified transformations (Barreñada et al., 2024). RF is well suited to mixed-type predictors and correlated laboratory features, and it integrates naturally with tree-based SHAP (TreeExplainer) to enable clinically interpretable global and patient-level explanations (Barreñada et al., 2024).

While ML models have been applied to infection-related tasks (e.g., recurrence or antimicrobial resistance prediction), evidence remains relatively limited for culture-independent pathogen differentiation in hospitalized UTI cohorts using only routinely available clinical and laboratory data. For example, a *Scientific Reports* study reported an AUC of 0.88 for identifying *E. coli* infections in elderly sepsis patients (Li et al., 2024). However, the target condition (sepsis) and clinical context differ substantially from UTIs, and the results are therefore not directly comparable. Similarly, a 2025 cohort study using 8,065 urinalysis and demographic records achieved an AUC of 0.79 for predicting overall urine culture positivity, but it was not designed to distinguish *E. coli* from other uropathogens among confirmed UTI patients (Sergounioti et al., 2025). These differences in outcome definition, population, and data sources underscore the need for task-specific models in hospitalized UTI settings.

In this study, we developed and internally validated an RF–based model to differentiate *E. coli*–associated UTIs from those caused by other uropathogens in hospitalized patients, based on retrospectively collected clinical data. We hypothesized that routinely available inflammatory and biochemical markers exhibit distinct profiles between pathogen groups, enabling clinically useful risk stratification prior to culture results. This model is intended to facilitate early, culture-independent decision support and potentially inform more timely and targeted clinical management in patients with suspected UTIs.

## Materials and methods

### Study design and data source

**Figure 1:**
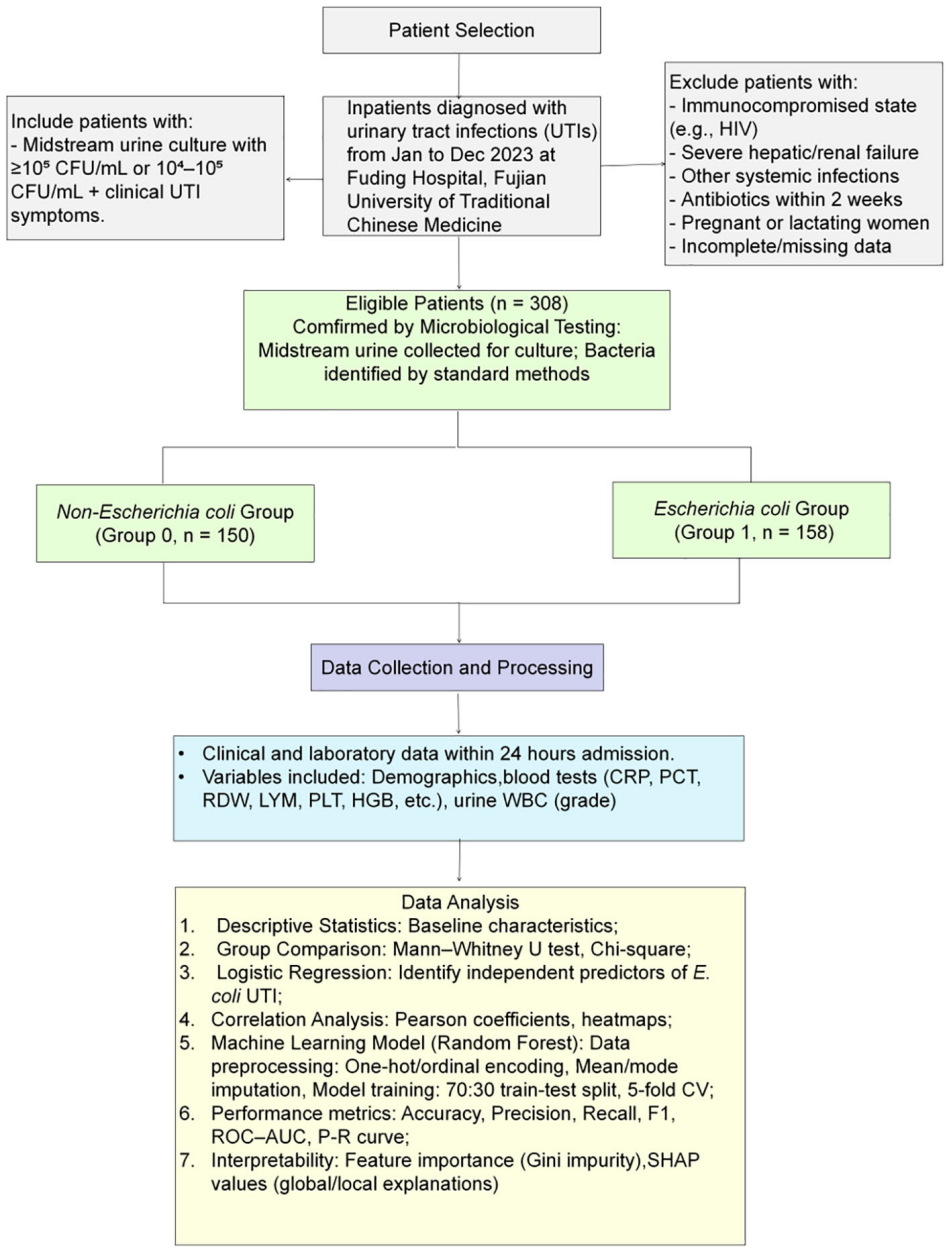
Study design and patient selection flowchart.

This retrospective cohort study was conducted at Fuding Hospital, Fujian University of Traditional Chinese Medicine. We consecutively screened hospitalized patients with a diagnosis of UTI and available urine culture results between January and December 2023. For patients with repeated admissions during the study period, only the first eligible admission was retained to avoid within-patient correlation. Records missing the primary outcome (urine culture–based pathogen group) were excluded; missing values in predictors were handled as described in the Data preprocessing section (imputation within the modeling pipeline). The overall study design and workflow are summarized in **Figure 1**.

Before analysis, the electronic medical record extract was pseudonymized (de-identified) by the hospital information team. All direct personal identifiers (e.g., name, national ID number, phone number, and address) were removed and each record was assigned a unique study code. The code key linking study codes to patient identities was stored separately by the hospital and was not accessible to the research team. Therefore, the analytic dataset contained no direct identifiers and included only variables required for this study.

The study protocol was approved by the Ethics Committee of Fuding Hospital, Fujian University of Traditional Chinese Medicine (Approval No. 2024015). All data were de-identified (anonymized) by the hospital information team prior to analysis, and the linkage key was not accessible to the research team, ensuring compliance with ethical and privacy standards. Due to the retrospective nature of the study, the requirement for written informed consent was waived by the ethics committee. The study was conducted in accordance with applicable local regulations and the Declaration of Helsinki.

Participants were classified into two groups based on urine culture results (midstream clean-catch specimens; catheter specimens were handled according to routine clinical practice, if applicable):

Non*-E. coli* group (Group 0, n = 150): Patients infected with uropathogens other than *E. coli*, including *Klebsiella pneumoniae*, *Acinetobacter baumannii*, *Enterococcus* spp., and other bacterial species.

*E. coli* group (Group 1, n = 158): Patients infected with *E. coli* identified as the predominant uropathogen.

Inclusion criteria comprised a positive urine culture with bacterial colony counts meeting standard clinical microbiology thresholds: ≥10^5^ colony-forming units (CFU)/mL, or 10^4^–10^5^ CFU/mL accompanied by clinical signs and symptoms consistent with UTIs. Exclusion criteria included: (1) immunocompromised status (e.g., human immunodeficiency virus (HIV) infection or long-term immunosuppressive therapy), (2) severe hepatic or renal failure, (3) disseminated malignancy, (4) concomitant infections outside the urinary tract, or (5) recent antibiotic use within 2 weeks before hospital admission to reduce culture-negative misclassification and biomarker distortion.

### Data collection and biomarker assessment

Demographic characteristics (age, sex) and laboratory test results were extracted from the electronic medical records. All laboratory analyses were performed in accordance with standard operating procedures for clinical diagnostics and were those obtained at/near the time of urine culture collection (index encounter).

### Blood and urine sample collection

Peripheral venous blood samples were collected under sterile conditions according to routine clinical practice as follows:

2.0 mL in EDTA tubes for complete blood count (CBC);

2.0 mL in citrate tubes for coagulation testing (e.g., D-dimer);

5.0 mL in plain tubes for serum biochemical analyses.

Urine specimens for urinalysis and microbial culture were collected using standard clinical procedures. Midstream clean-catch urine was preferred; when clean-catch was not feasible (e.g., catheterized patients), urine was collected via catheter specimen according to hospital protocol. Samples were obtained in sterile containers and transported to the microbiology laboratory promptly for analysis.

### Microbial culture and identification

Urine cultures were performed by inoculating 1 μL of well-mixed urine onto 5% sheep blood agar plates using a calibrated loop. Samples were streaked in a standardized pattern and incubated at 35–37°C under aerobic conditions for 18–24 hours, with extended incubation up to 48 hours when needed. Where appropriate, MacConkey agar was used in parallel for differentiation of Gram-negative bacilli. Significant growth was defined as ≥10^5^ CFU/mL, or 10^4^–10^5^ CFU/mL in symptomatic patients. Urine specimens were primarily midstream clean-catch; catheter specimens were processed according to the same laboratory protocol when applicable.

Preliminary identification was based on colony morphology and Gram staining. Definitive species-level identification was conducted using the VITEK MS mass spectrometry system (bioMérieux, France), following the manufacturer’s protocols. Internal quality control was maintained using standard reference strains, such as *Escherichia coli* ATCC 25922.

### Biomarker measurements

#### Inflammatory markers

PCT levels were measured using electrochemiluminescence immunoassay (MCL60, Rismay, Nanjing, China); CRP levels were measured using latex-enhanced immunoturbidimetry (BC-7500CS, Mindray, Shenzhen, China).

#### Hematological parameters

CBC indices, including neutrophils, LYM, hemoglobin (HGB), red cell distribution width (RDW), and platelets (PLT) were analyzed using an automated hematology analyzer (BC-7500CS, Mindray, Shenzhen, China).

#### Coagulation markers

D-dimer levels were quantified by immunoturbidimetry (ExC810, Mindray, Shenzhen, China).

#### Biochemical markers

Serum levels of ALB, blood urea nitrogen (BUN), glucose (GLU), total bilirubin (TBIL), high-density lipoprotein (HDL), total cholesterol (CHO), ALT, and aspartate aminotransferase (AST) were measured using the AU5800 automated biochemical analyzer (Beckman Coulter, USA).

#### Urinalysis

Urinary leukocyte (urinary WBC) counts were assessed using an automated urine analyzer (UF-500i, Sysmex, Japan) and recorded as semi-quantitative grades (ordinal categories) according to the analyzer output, consistent with the modeling strategy.

### Random forest modeling

Random forest modeling was performed using Python 3.7. The computational environment incorporated the following libraries: NumPy (v1.21.0) for numerical operations, Pandas (v1.3.0) for data manipulation, Scikit-learn (v0.24.2) for machine learning implementation and performance evaluation, and Matplotlib (v3.4.2) for visualization.

### Data preprocessing

#### Missing data handling

Missing values in predictors were imputed using the median within the modeling pipeline (SimpleImputer, strategy = “median”). Imputation parameters were learned from the training data only; within five-fold cross-validation, the imputer was fit on each fold’s training subset and applied to its corresponding validation subset, and the final imputer was then refit on the full training set and applied to the held-out test set to prevent information leakage. Variable-wise missingness rates are reported in **Supplementary Table S1**.

#### Categorical encoding

Sex was coded as a single binary indicator, and urinary leukocyte grade (Urinary_WBC) was treated as an ordinal variable. No nominal categorical predictors were included in the final model; therefore, one-hot encoding was not required.

#### Feature dimensionality

In total, the RF model used 19 predictors (17 continuous laboratory variables plus Sex and urinary leukocyte grade), yielding a feature matrix of 308 × 19 prior to the train/test split.

#### Model validation

The dataset was split once into a training set (70%) and a held-out test set (30%) using stratification by the outcome. Five-fold stratified cross-validation was performed within the training set for hyperparameter tuning and internal performance estimation. All final discrimination, calibration, and operating-point metrics were reported on the held-out test set.

### Model evaluation metrics

#### Classification performance

Model performance was assessed using standard classification metrics derived from the confusion matrix, including true positives (TP), false positives (FP), true negatives (TN), and false negatives (FN). The following indices were calculated:

Precision = TP/(TP + FP).

Recall (Sensitivity) = TP/(TP + FN).

F1-score = 2 × (Precision × Recall)/(Precision + Recall).

ROC curves were plotted to evaluate the model’s discriminative capacity. The AUC was computed as a global measure of diagnostic discrimination. Uncertainty for AUC, AP, Brier score, and operating-point metrics was quantified by bootstrap resampling of the held-out test set (1,000 iterations), with 95% CIs defined by the 2.5th and 97.5th percentiles.

In addition, Precision–Recall (P–R) curves were generated to characterize model performance in identifying the positive class (*E. coli* infection). The AP score was used to summarize the trade-off between precision and recall across thresholds. Although class proportions were similar in this cohort, PR-based evaluation remains informative for assessing positive-class performance and threshold-dependent trade-offs.

Clinically meaningful operating points were reported at the default threshold (0.50) and at a threshold selected on the training set using the Youden index computed from five-fold out-of-fold predicted probabilities, which was then applied to the held-out test set.

#### Model calibration

Calibration was evaluated using the Brier score (mean squared error between predicted probabilities and observed outcomes) and the calibration slope, estimated by fitting a logistic regression model of the outcome on the logit-transformed predicted probabilities. A calibration curve (reliability diagram) was generated to visually assess agreement between predicted and observed risks.

### Statistical analysis

All statistical analyses were performed using SPSS software (version 22.0, IBM Corp., Armonk, NY, USA). The distribution of continuous variables was assessed using the Shapiro–Wilk test. Variables conforming to normal distribution were expressed as mean ± standard deviation (SD). Non-normally distributed variables were reported as median with interquartile range (IQR) and analyzed using the Mann–Whitney U test. Categorical variables were compared using the chi-square (χ²) test.

To identify independent predictors of *E. coli* infection, univariate logistic regression was first conducted. Variables with a p-value < 0.20 were subsequently entered into a multivariable logistic regression model using a backward stepwise elimination strategy. Adjusted odds ratios (ORs) and corresponding 95% confidence intervals (CIs) were reported. All statistical tests were two-tailed, and a P value < 0.05 was considered statistically significant.

### Random forest model and hyperparameter tuning

A RF classifier (scikit-learn) was trained to distinguish between *E. coli* (Group = 1) and non–*E. coli* (Group = 0) UTIs. Missing predictor values were imputed using a median strategy within a pipeline. Imputation parameters were estimated from the training set only, and then applied to the held-out test set to prevent information leakage. The dataset was split once using a stratified 70/30 train-test split (random_state = 42).

Hyperparameter tuning was conducted on the training set using RandomizedSearchCV with 5-fold stratified cross-validation, optimizing ROC-AUC. The candidate search space included: n_estimators ∈ {300, 500, 800, 1200}, max_depth ∈ {None, 3, 5, 7, 10, 15}, min_samples_split ∈ {2, 5, 10, 20}, min_samples_leaf ∈ {1, 2, 3, 5, 8}, and max_features ∈ {“sqrt”, “log2”, 0.3, 0.5, 0.8}. To address potential class imbalance, the class_weight parameter was set to “balanced”. The final RF hyperparameters selected by RandomizedSearchCV were: n_estimators = 300, max_depth = 15, min_samples_split = 10, min_samples_leaf = 8, max_features = log2, with class_weight = “balanced” and random_state = 42.

### Baseline models and robustness checks

To contextualize the RF model and reduce the risk of model-specific findings, we benchmarked RF against commonly used baseline classifiers trained on the same feature set and data split. These baselines included (i) regularized logistic regression (L2-penalized), (ii) support vector machine (SVM), and (iii) gradient boosting (tree-based boosting). For all models, missing predictors were imputed using the same median-imputation strategy within a training-only pipeline to prevent information leakage; continuous features were standardized for linear models (logistic regression/SVM). Hyperparameters were tuned using stratified cross-validation within the training set, and final performance was reported on the held-out stratified test set using the same discrimination (ROC-AUC, PR-AUC) and calibration metrics (Brier score; calibration intercept/slope).

### Feature importance and SHAP analysis

To interpret the RF model, we used SHAP to quantify each feature’s contribution to the predicted probability of *E. coli* at the individual level. SHAP values were computed using TreeExplainer (TreeSHAP) following the framework described by Lundberg et al (Lundberg et al., 2020), which provides consistent additive attributions for tree-based models and can reflect nonlinear and interaction-driven effects. Global importance was summarized as the mean absolute SHAP value computed on the held-out test set, and signed SHAP summary (beeswarm) plots were used to visualize directionality and heterogeneity. SHAP analyses were conducted using the Python SHAP package (v0.45.1).

## Results

### Normality testing of variables

The Shapiro–Wilk test revealed that most continuous variables, including CRP, ALT, AST, PCT, and D-dimer, deviated from a normal distribution in both groups (all P < 0.05). Only HGB) and ALB in the non-*E. coli* group demonstrated normal distribution (P = 0.309 and 0.773, respectively). Accordingly, non-parametric tests (e.g., Mann–Whitney U test) were employed for intergroup comparisons of non-normally distributed variables.

### Distribution of microorganisms

**Figure 2:**
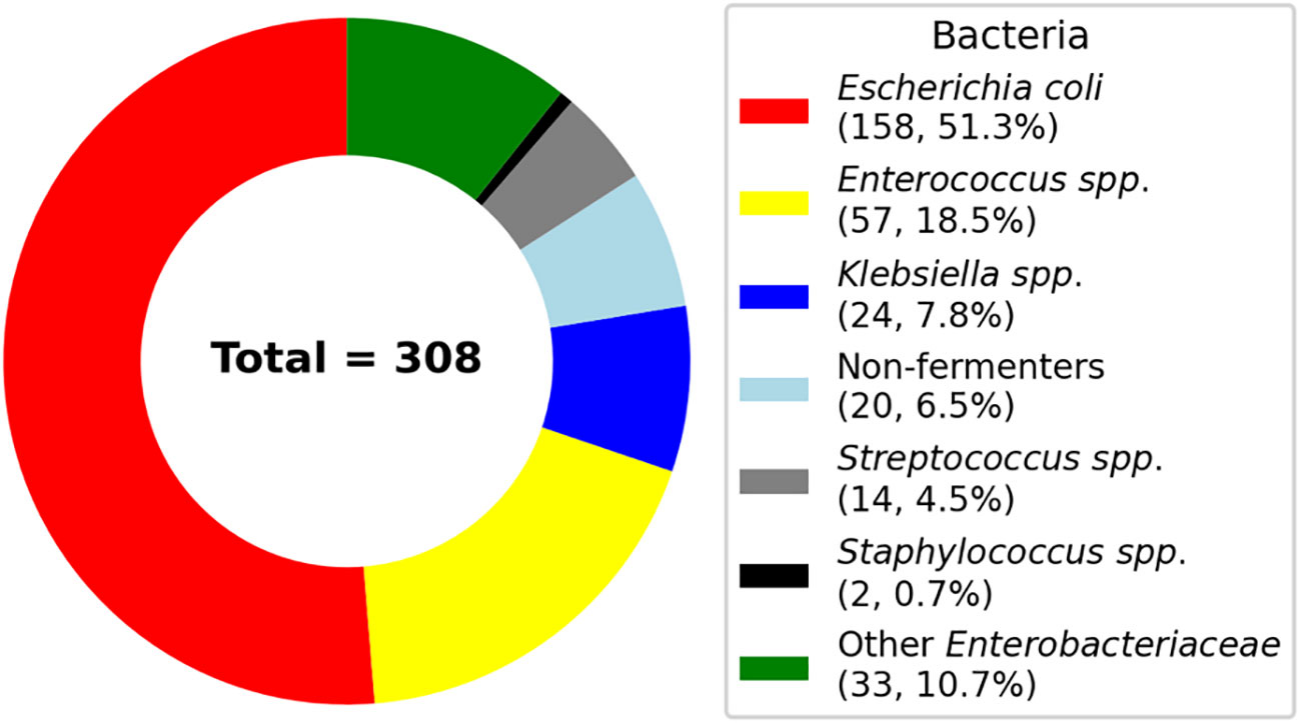
Distribution of bacterial isolates in patients with urinary tract infections: The donut chart illustrates the proportional representation of 308 isolates across taxonomic groups.

A total of 308 bacterial isolates were identified from patients with UTIs. As shown in **Figure 2**, *E. coli* was the most prevalent pathogen, comprising 158 isolates (51.3%), followed by *Enterococcus* spp. (57, 18.5%) and *Klebsiella* spp. (24, 7.8%). Other detected organisms included non-fermenting Gram-negative bacilli (20, 6.5%), *Streptococcus* spp. (14, 4.5%), *Staphylococcus* spp. (2, 0.7%), and other members of the *Enterobacteriaceae* family (33, 10.7%). A detailed breakdown of bacterial species and their respective proportions is presented in **Table 1**.

**Table 1:** Distribution of bacterial isolates identified from midstream urine samples (n = 308).

No.	Bacterial category	Species	Count	Percentage (%)
1	*Escherichia coli* (Total = 158)	–	158	51.30
2	*Enterococcus* spp. (Total = 57)	*Enterococcus faecium*	32	10.39
		*Enterococcus faecalis*	25	8.12
3	*Klebsiella* spp. (Total = 24)	*Klebsiella pneumoniae*	21	6.82
		*Citrobacter braakii*	2	0.65
		*Citrobacter freundii*	1	0.33
4	Non-fermenters Gram negative bacilli (Total = 20)	*Acinetobacter baumannii*	7	2.27
		*Stenotrophomonas maltophilia*	7	2.27
		*Pseudomonas aeruginosa*	5	1.62
		*Sphingomonas paucimobilis*	1	0.33
5	*Streptococcus agalactiae* (Total = 14)	–	14	4.55
6	*Staphylococcus* spp. (Total = 2)	–	2	0.65
7	Other *Enterobacteriaceae* (Total = 33)	*Morganella morganii*	10	3.25
		*Proteus mirabilis*	10	3.25
		*Enterobacter cloacae*	5	1.62
		*Hafnia alvei/Edwardsiella tarda*	4	1.30
		*Serratia marcescens*	3	0.97
		*Salmonella* spp.	1	0.33
	Total	–	308	100.00

Bacterial isolates were obtained from midstream urine specimens. Standard culture techniques were applied with internal quality controls using reference strains.

### Baseline characteristics and laboratory findings

A total of 308 patients were included: 150 in the non-*E. coli* group and 158 in the *E. coli* group. No significant difference was observed in median age between the groups (68.0 vs. 66.0 years, P = 0.301). However, gender distribution differed significantly, with a higher proportion of females in the *E. coli* group (74.3% vs. 44.7%, P < 0.001) (**Table 2**).

**Table 2:** Demographic characteristics of patients in the *Escherichia coli* and non-*E. coli* urinary tract infection groups.

Variable	Non*-E. coli* group (n = 150)	*E. coli* group (n = 158)	P value
Age (Year)	68.0 (57.8 - 77.0)	66.0 (55.8 - 75.0)	0.301
Age range	22.0 - 88.0	23.00 - 91.0	
Gender, n (%)			<0.001
Female	69 (46.0)	117 (74.1)	
Male	81 (54.0)	41 (25.9)	

Data are shown as median (interquartile range, IQR) for age and n (%) for categorical variable. Group comparisons were performed using the Mann–Whitney U test for non-normally distributed continuous data and the chi-square test (χ²) for categorical variable.

Compared to the non -*E. coli* group, the *E. coli* group exhibited significantly higher values of HGB (P = 0.004), LYM (P = 0.003), ALT (P = 0.001), and ALB (P = 0.021). In contrast, RDW (P = 0.016) and D-dimer levels (P = 0.014) were lower in the *E. coli* group (**Table 3**).

**Table 3:** Comparison of laboratory parameters between the *Escherichia coli* and non-*E. coli* urinary tract infection groups.

Variable	Non*-E. coli* group (n = 150)	*E. coli* group (n = 158)	P value
HGB (g/L)	112.05 ± 23.26	121.50 (108.75 - 133.00)	0.004
RDW (%)	14.03 (13.10 - 14.90)	13.90 (12.90 - 14.03)	0.016
Neutrophils (×10^9^/L)	4.28 (3.20 - 6.16)	3.89 (3.10 - 5.43)	0.382
LYM (×10^9^/L)	1.33 (0.943 - 1.78)	1.58 (1.17 -1.92)	0.003
Platelets (×10^9^/L)	223.00 (180.00 - 292.25)	237.50 (189.50 - 303.25)	0.354
C-reactive protein (mg/L)	10.57 (4.90 - 26.78)	6.63 (4.90 - 17.93)	0.073
Procalcitonin (ng/mL)	0.11 (0.05 - 0.78)	0.11 (0.07 - 0.78)	0.426
ALT (U/L)	15.00 (9.00 - 27.00)	20.00 (13.00 - 31.00)	0.001
AST (IU/L)	20.00 (15.00 - 28.00)	21.00 (17.00 - 28.00)	0.253
ALB (g/L)	36.07 ± 5.47	37.90 (33.80 - 41.73)	0.021
TBIL (μmol/L)	9.20 (6.18 - 12.20)	8.75 (6.70 -11.93)	0.747
BUN (mmol/L)	5.16 (3.78 - 8.53)	4.90 (3.90 - 6.64)	0.316
HDL (mmol/L)	1.02 (0.82 - 1.15)	1.01 (0.82 -1.16)	0.886
Glucose (mmol/L)	6.23 (4.97 - 7.79)	6.28 (5.09 - 7.74)	0.839
Cholesterol (mmol/L)	4.21 (3.39 - 4.63)	4.21 (3.49 - 4.84)	0.198
D-dimer (ng/mL)	1.17 (0.61 - 1.71)	0.80 (0.41 - 1.71)	0.014

Data are presented as median (interquartile range, IQR). Non-normally distributed variables were analyzed using the Mann–Whitney U test. HGB, hemoglobin; LYM, lymphocytes; RDW, red cell distribution width; ALT, alanine aminotransferase; AST, aspartate aminotransferase; ALB, albumin; BUN, blood urea nitrogen; TBIL, total bilirubin; HDL, high-density lipoprotein cholesterol.

### Logistic regression analysis of risk factors

As shown in **Table 4**, univariate logistic regression analysis identified eight variables significantly associated with the presence of *E. coli* UTI, including HGB, RDW, LYM, ALT, ALB, BUN, sex, and urinary WBC (grade 1). These variables were subsequently entered into a multivariate logistic regression model to determine independent predictors (**Table 5**).

**Table 4:** Univariate logistic regression analysis of potential predictors for *Escherichia coli* urinary tract infection.

Variable	Reference group	P value	OR (95% CI)
Age (year)		0.351	0.993 (0.977 - 1.008)
Hemoglobin (g/L)		**0.009**	1.014 (1.003 - 1.025)
RDW (%)		**0.061**	0.879 (0.768 - 1.006)
Neutrophils (×10^9^/L)		0.264	0.962 (0.900 - 1.029)
LYM (×10^9^/L)		**0.006**	1.730 (1.168 - 2.563)
Platelets (×10^9^/L)		0.555	1.001 (0.998 - 1.003)
CRP (mg/L)		0.246	0.997 (0.991-1.002)
PCT (ng/mL)		0.921	0.999 (0.980 - 1.019)
ALT (U/L)		**0.024**	1.014 (1.002 - 1.027)
AST (U/L)		0.293	1.004 (0.996 - 1.013)
ALB (g/L)		**0.045**	1.043 (1.001 - 1.088)
TBIL (μmol/L)		0.236	1.020 (0.987 - 1.055)
BUN (mmol/L)		**0.064**	0.955 (0.909 - 1.003)
HDL (mmol/L)		0.990	0.996 (0.517 - 1.917)
Glucose (mmol/L)		0.432	1.023 (0.966 - 1.084)
D-dimer		0.491	0.976 (0.912 - 1.045)
Cholesterol (mmol/L)		0.127	1.151 (0.961-1.380)
Sex	Female	**<0.001**	0.299 (0.185 - 0.482)
Urinary WBC (Grade 1)	Grade 0	0.002	2.857 (1.462 - 5.583)
Urinary WBC (Grade 2)	Grade 0	0.912	0.964 (0.505 - 1.843)
Urinary WBC (Grade 3)	Grade 0	0.126	1.564 (0.881 - 2.774)

Univariate logistic regression was conducted to evaluate associations between clinical variables and the likelihood of *E. coli* urinary tract infection. OR > 1 indicate increased risk, and OR < 1 indicate decreased risk. Variables with P < 0.20 were considered candidates for multivariate analysis. SE, standard deviation; OR, odds ratio; CI, confidence interval; HGB; hemoglobin; LYM, lymphocytes; RDW, red cell distribution width; CRP, C-reactive protein; PCT, procalcitonin; ALT, alanine aminotransferase; AST, aspartate aminotransferase; ALB, albumin; TBIL, total bilirubin; BUN, blood urea nitrogen; HDL, high-density lipoprotein; WBC, white blood cells.

Bold values indicate statistical significance (P <. 0.05).

**Table 5:** Multivariable logistic regression analysis for independent predictors of *Escherichia coli* urinary tract infects.

Variable	Reference group	P-value	OR (95% CI)
RDW (%)		0.095	0.880 (0.758 - 1.022)
LYM (×10^9^/L)		**0.016**	1.690 (1.104 - 2.587)
ALT (U/L)		0.057	1.013 (1.000 - 1.027)
Sex	Female	**<0.001**	0.299 (0.181 - 0.496)
Urinary WBC (Grade 1)	Grade 0	**0.002**	3.100 (1.514 - 6.346)
Urinary WBC (Grade 2)	Grade 0	0.771	1.109 (0.553 - 2.224)
Urinary WBC (Grade 3)	Grade 0	0.080	1.738 (0.936 - 3.226)
Constant		0.466	2.348

SE, standard deviation; OR, odds ratio; CI, confidence interval; RDW, red cell distribution width; LYM, lymphocytes; ALT, alanine aminotransferase; WBC, white blood cells.

Bold values indicate statistical significance (P <. 0.05).

The multivariable analysis revealed three statistically significant independent predictors of *E. coli* UTIs: LYM (OR = 1.690, P = 0.016); sex (male) (OR = 0.299, P < 0.001); Urinary WBC (Grade 1) (OR = 3.100, P = 0.002) (**Table 5**).

These results indicate that females are more likely to develop *E. coli*-associated UTIs. Additionally, elevated LYM and the presence of urinary WBC (grade 1) significantly increased the risk of *E. coli* UTIs.

### Correlation analysis

**Figure 3:**
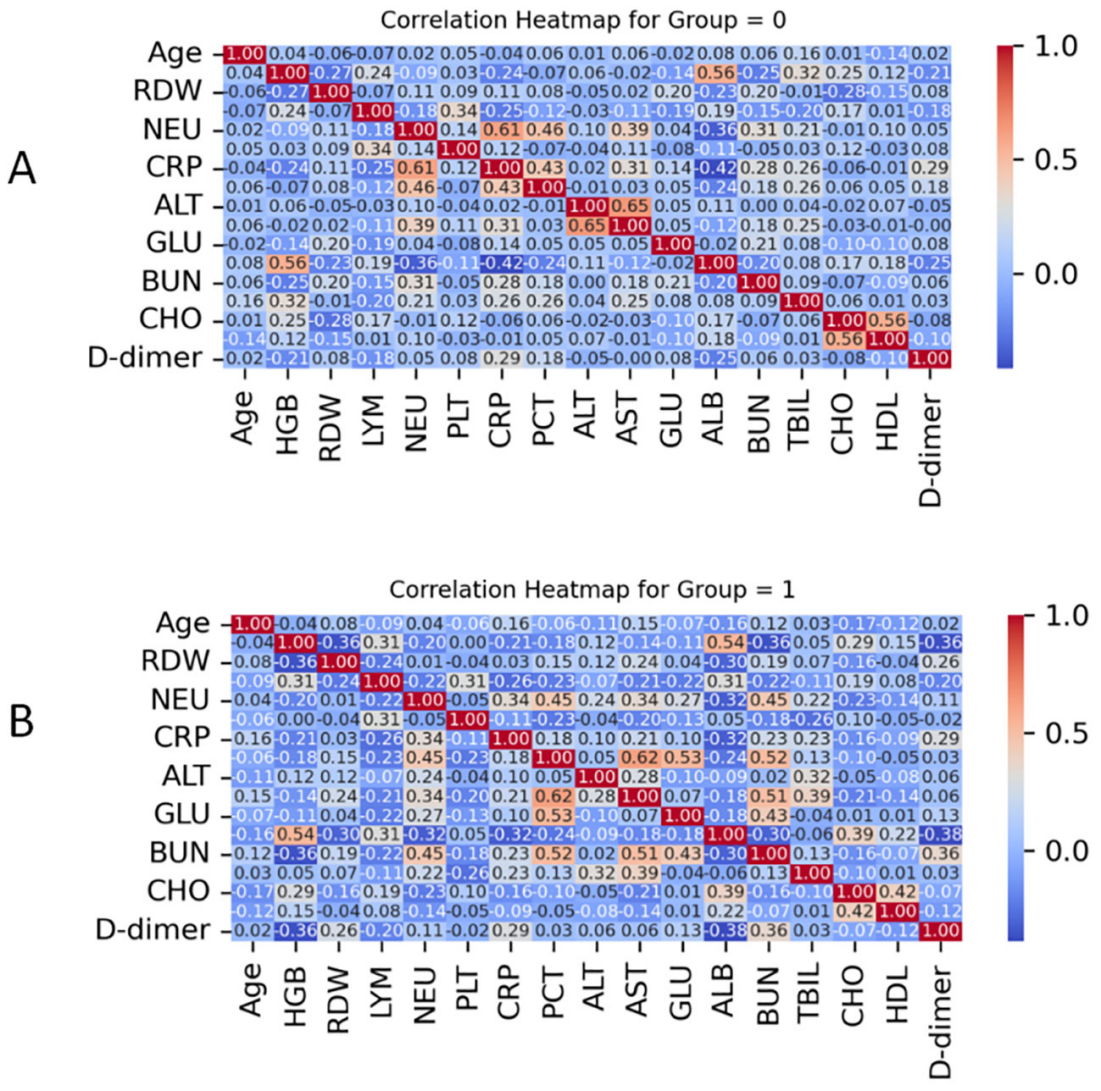
Pearson correlation heatmaps of clinical variables in the two groups: **(A)** Non-*Escherichia coli* group (Group 0); **(B)***Escherichia coli* group (Group 1): The heatmaps illustrate pairwise Pearson correlation coefficients (r) among clinical and biochemical variables. Color gradients reflect the strength and direction of associations, from strong positive (red, r = 1.0) to strong negative (blue, r = −1.0).

Pearson correlation heatmaps were constructed separately for the non-*E. coli* group (Group 0) and the *E. coli* group (Group 1) to examine inter-variable associations (**Figures 3A, B**).

In the non-*E. coli* group (Group 0), most variable pairs demonstrated weak correlations (|r| < 0.3). Notable moderate correlations were observed between CRP and neutrophils (r = 0.61), between HDL and CHO (r = 0.56), and RDW and HGB (r = 0.56).

In contrast, Group 1 exhibited more pronounced inter-variable relationships. A strong correlation was observed between PCT and AST (r = 0.62), while a moderate correlation was noted between PCT and GLU (r = 0.53).

These distinct patterns suggest that the systemic inflammatory and metabolic responses may differ between *E. coli* and non-*E. coli* UTIs.

### Confusion matrix of the random forest model

**Figure 4:**
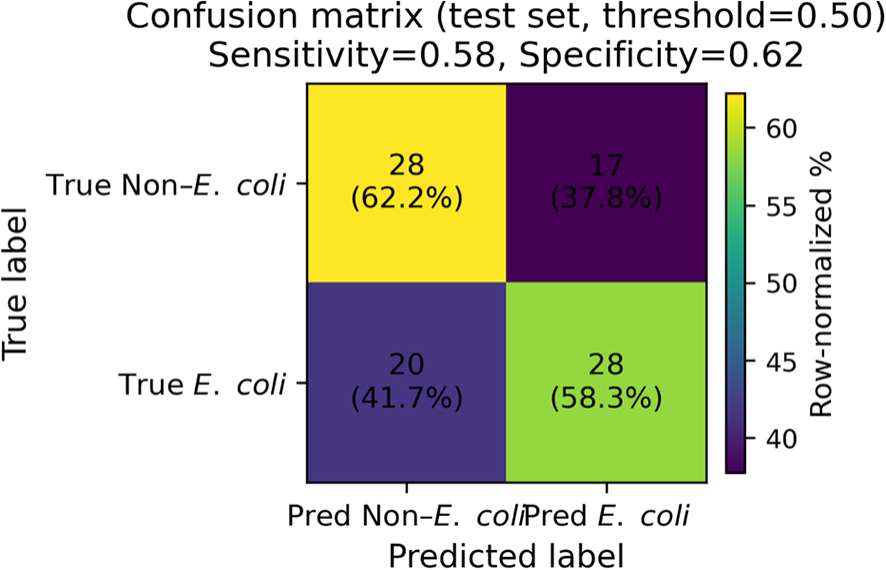
Confusion matrix of the Random Forest model on the held-out test set (n = 93) using a fixed decision threshold of 0.50 for predicting *E. coli* (Group = 1). Cells show counts with row-normalized percentages in parentheses (each row sums to 100%), enabling interpretation of error patterns within each true class. The title reports sensitivity (true positive rate) and specificity (true negative rate) computed on the test set at the same threshold.

The confusion matrix for the random forest model on the test set is shown in **Figure 4**. The model correctly identified 30 patients in the non-*E. coli* group and 29 in the *E. coli* group. Misclassifications included 15 false positives (non-*E. coli* cases incorrectly predicted as *E. coli*) and 19 false negatives. This distribution reflects a relatively balanced classification performance, though slight misclassification bias toward both classes was observed.

### Performance of the random forest model

Because the final predictor dimensionality was limited (P = 19) relative to the sample size (n = 308), the model was not exposed to high-dimensional one-hot expansion. Model complexity was further controlled by cross-validated hyperparameter tuning within the training set and evaluated on a held-out test set.

On the held-out test set (n = 93), the RF model achieved an overall accuracy of 0.63. Class-wise precision/recall/F1 were 0.61/0.67/0.64 for the non–*E. coli* group and 0.66/0.60/0.63 for the *E. coli* group (**Supplementary Table S2**).

### Discrimination and calibration are summarized

The model showed moderate discrimination with a ROC-AUC of 0.66 (95% CI 0.54–0.77) and a PR-AUC of 0.66 (95% CI 0.54–0.81). Calibration was acceptable, with a Brier score of 0.233 (95% CI 0.210–0.255) and a calibration slope of 1.130 (intercept 0.124) (**Table 6**).

**Table 6:** Discrimination and calibration on the held-out test set (n=93).

Metric	Estimate (95% CI)
ROC-AUC	0.66 (0.54-0.77)
PR-AUC (Average precision)	0.66 (0.54-0.81)
Brier score	0.233 (0.210-0.255)
Calibration intercept	0.124
Calibration slope	1.130

CI, confidence interval; ROC-AUC, area under the receiver operating characteristic curve; PR-AUC, area under the precision–recall curve (average precision, AP). ROC-AUC and PR-AUC were calculated on the held-out test set (n = 93). The Brier score reflects the mean squared error between predicted probabilities and observed outcomes (lower is better). Calibration intercept and slope were estimated by regressing the observed outcome on the logit of predicted probabilities (intercept ≈ 0 and slope ≈ 1 indicate ideal calibration). 95% confidence intervals for AUC, PR-AUC, and Brier score were obtained by bootstrap resampling of the held-out test set (1,000 iterations).

### Clinically meaningful operating points are reported

At the default threshold of 0.50, the model achieved an accuracy of 0.65 (95% CI 0.55–0.74) with sensitivity 0.62 (0.49–0.76) and specificity 0.67 (0.52–0.80) (**Table 7**). Using the Youden threshold determined on the training set (0.476) yielded sensitivity 0.65 (0.52–0.79) and specificity 0.60 (0.44–0.74).

**Table 7:** Clinically meaningful operating points on the held-out test set (n=93, 95% CI).

Threshold	Accuracy	Sensitivity	Specificity	PPV	NPV	F1
0.50 (default)	0.65 (0.55–0.74)	0.62 (0.49–0.76)	0.67 (0.52-0.80)	0.67 (0.52–0.80)	0.62 (0.49–0.76)	0.65 (0.52–0.75)
0.476 (Youden; training OOF)	0.62 (0.53–0.72)	0.65 (0.52–0.79)	0.60 (0.44 - 0.74)	0.63 (0.50–0.76)	0.61 (0.46–0.76)	0.64 (0.52–0.74)

PPV, positive predictive value; NPV, negative predictive value; CI, confidence interval; OOF, out-of-fold. Operating-point metrics were evaluated on the held-out test set (n = 93). The default threshold was 0.50. The Youden threshold (0.476) was selected on the training set using 5-fold out-of-fold predicted probabilities by maximizing Youden’s J (sensitivity + specificity − 1), and then applied unchanged to the test set. 95% confidence intervals were obtained by bootstrap resampling of the held-out test set (1,000 iterations).

### ROC and precision–recall curve analysis

**Figure 5:**
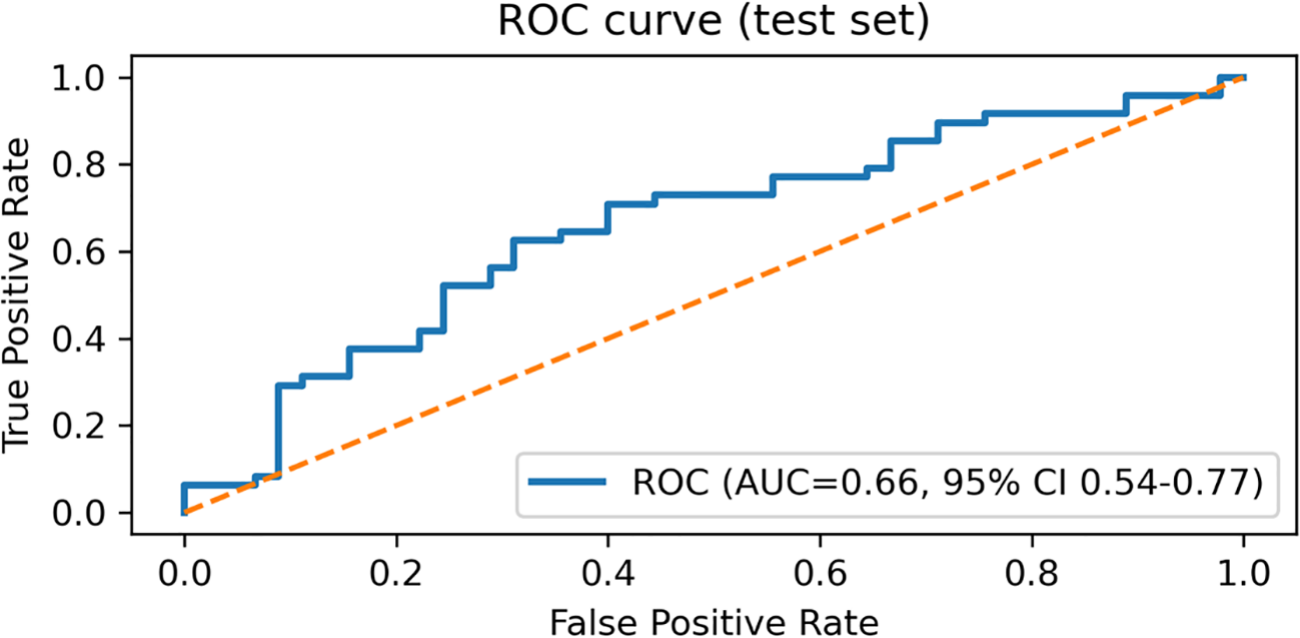
Receiver operating characteristic (ROC) curve of the Random Forest model on the held-out test set (n = 93), constructed from predicted probabilities for *E. coli* (Group = 1). The area under the ROC curve (ROC-AUC) is reported with a 95% confidence interval estimated by bootstrap resampling (1,000 iterations) of the test set.

**Figure 6:**
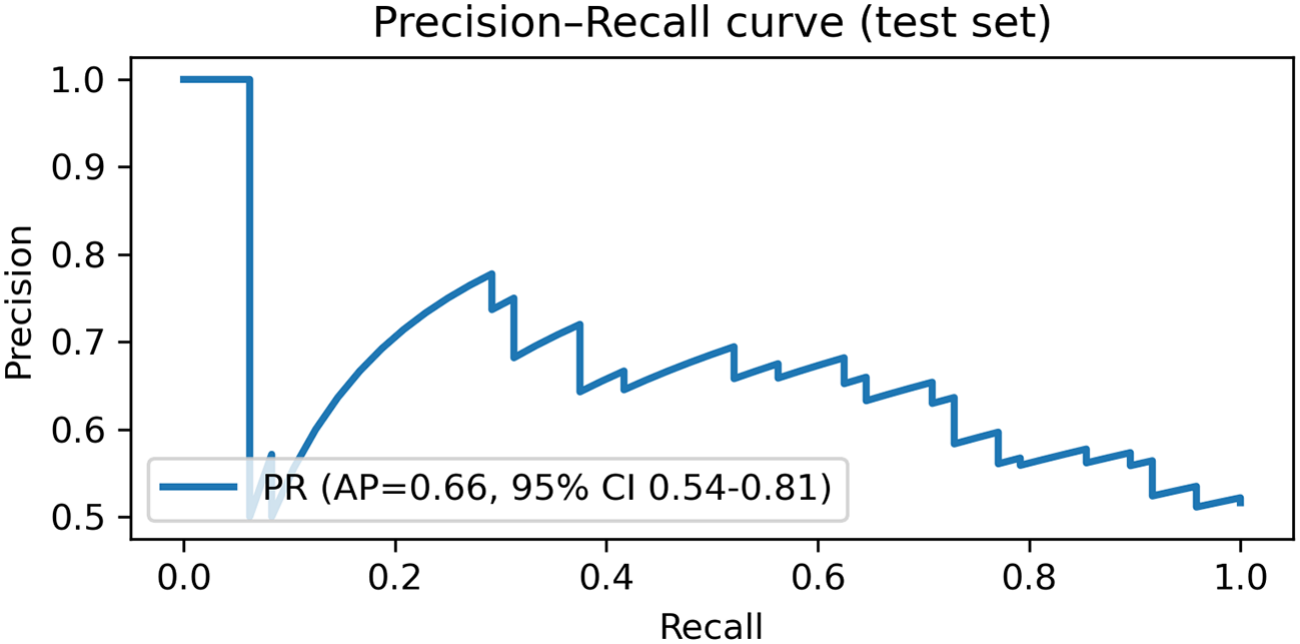
Precision–recall (PR) curve of the Random Forest model on the held-out test set (n = 93), constructed from predicted probabilities for *E. coli* (Group = 1). The area under the PR curve (PR-AUC/average precision) is reported with a 95% bootstrap confidence interval (1,000 iterations).

The diagnostic performance of the RF model was further evaluated using the ROC curve and the precision–recall (P–R) curve (**Figures 5**, **6**). As shown in **Figure 5**, the ROC curve remained above the diagonal reference line across most thresholds, indicating discriminative ability beyond chance. The corresponding ROC-AUC, with the 95% confidence interval, is reported in **Table 6**. As shown in **Figure 6**, the P–R curve was generally above the baseline precision determined by the positive-class prevalence, supporting clinically meaningful precision–recall trade-offs. The corresponding PR-AUC (average precision), with 95% confidence interval, is summarized in **Table 6**.

### Benchmarking against baseline models

The RF model’s performance was benchmarked against several standard baselines, including regularized logistic regression, SVM, and gradient boosting, using the same stratified held-out evaluation. The RF model demonstrated comparable performance to these baselines, with ROC-AUC values ranging from 0.59 to 0.66 and PR-AUC values from 0.61 to 0.66 (**Supplementary Table S3**). Notably, the discrimination remained moderate across all methods, justifying the decision to report conservative performance estimates derived from stratified evaluation rather than potentially optimistic non-stratified splits.

### Feature stability checks

Further stability checks were conducted to compare the top features selected by each model. The overlap of the top-10 features, measured by the Jaccard index, and the rank correlation of feature importance were assessed to evaluate the consistency of the selected biomarkers across different models. **Supplementary Figure S1** presents the Jaccard index of top-10 features across the models, while **Supplementary Figure S2** shows the Spearman rank correlation between the importance rankings of these features.

### Calibration performance

**Figure 7:**
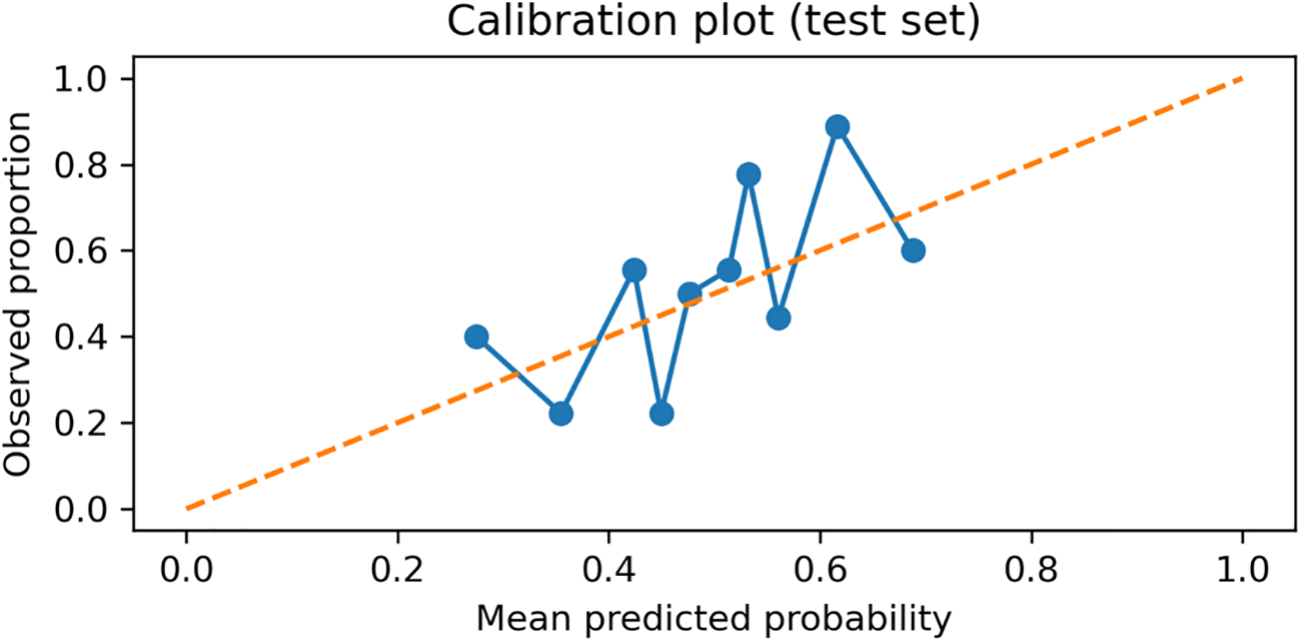
Calibration plot (reliability diagram) of the Random Forest model on the held-out test set (n = 93). The dashed line indicates perfect calibration; points represent the observed proportion of *E. coli* (Group = 1) within quantile-based bins of predicted probabilities, plotted against the mean predicted probability in each bin.

The model showed acceptable calibration on the held-out test set (Brier score = 0.233; calibration intercept = 0.124; calibration slope = 1.130; **Table 6**). The calibration curve is shown in **Figure 7**.

### Feature importance

**Figure 8:**
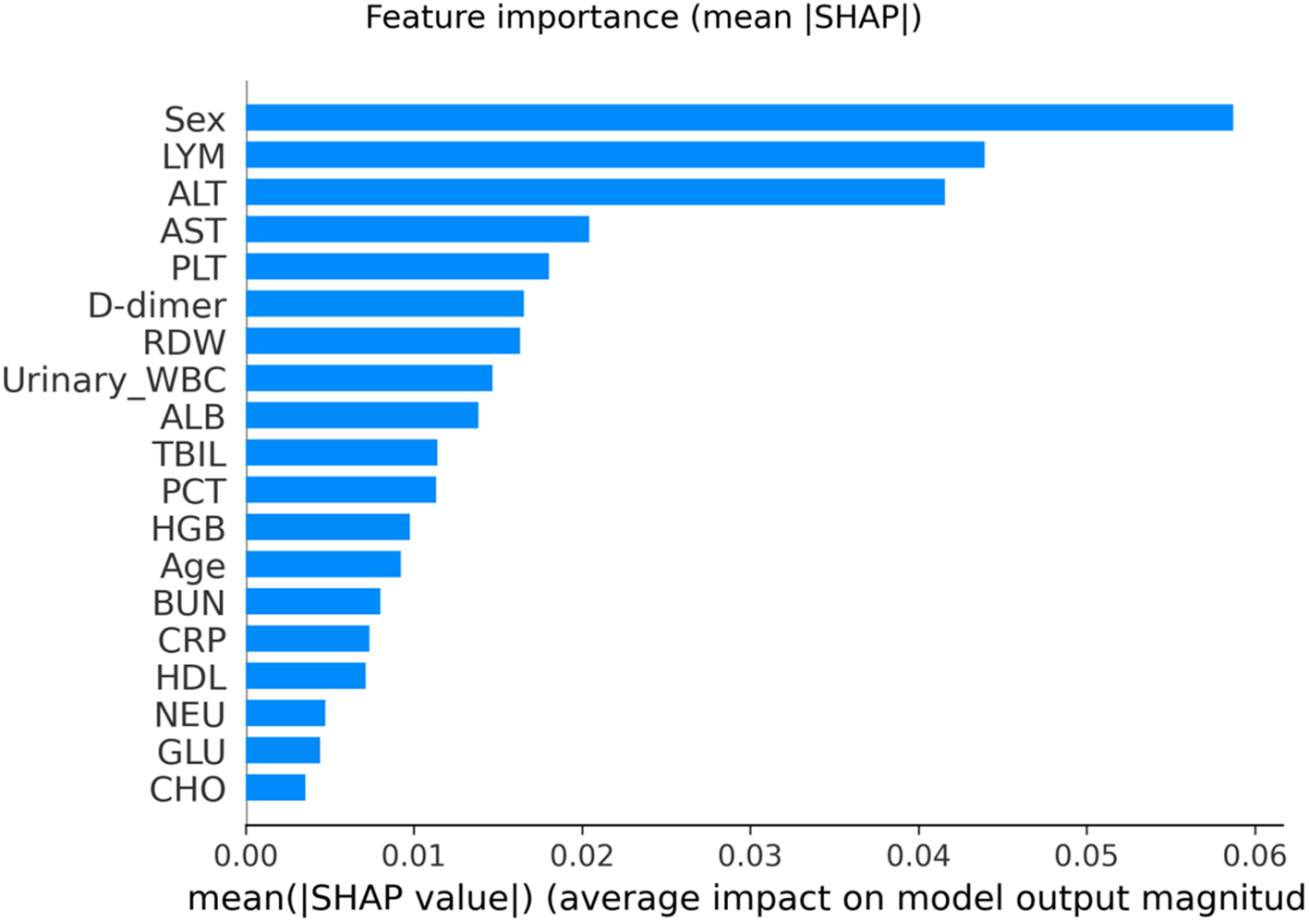
Global feature importance based on mean absolute SHAP values (mean |SHAP|) for the Random Forest model, computed on the held-out test set (n = 93). Larger mean |SHAP| indicates a greater average contribution magnitude of the feature to the model’s prediction for *E. coli* (Group = 1). RDW, red cell distribution width; CRP, C-reactive protein; PCT, procalcitonin; ALT, alanine aminotransferase; AST, aspartate aminotransferase; TBIL, total bilirubin; BUN, blood urea nitrogen; HDL, high-density lipoprotein; WBC, white blood cells.

Model interpretability was assessed using SHAP. Global SHAP ranking (mean |SHAP| on the test set) identified Sex, LYM, and ALT as the most influential predictors contributing to the model’s output for *E. coli* classification (**Figure 8**).

**Figure 9:**
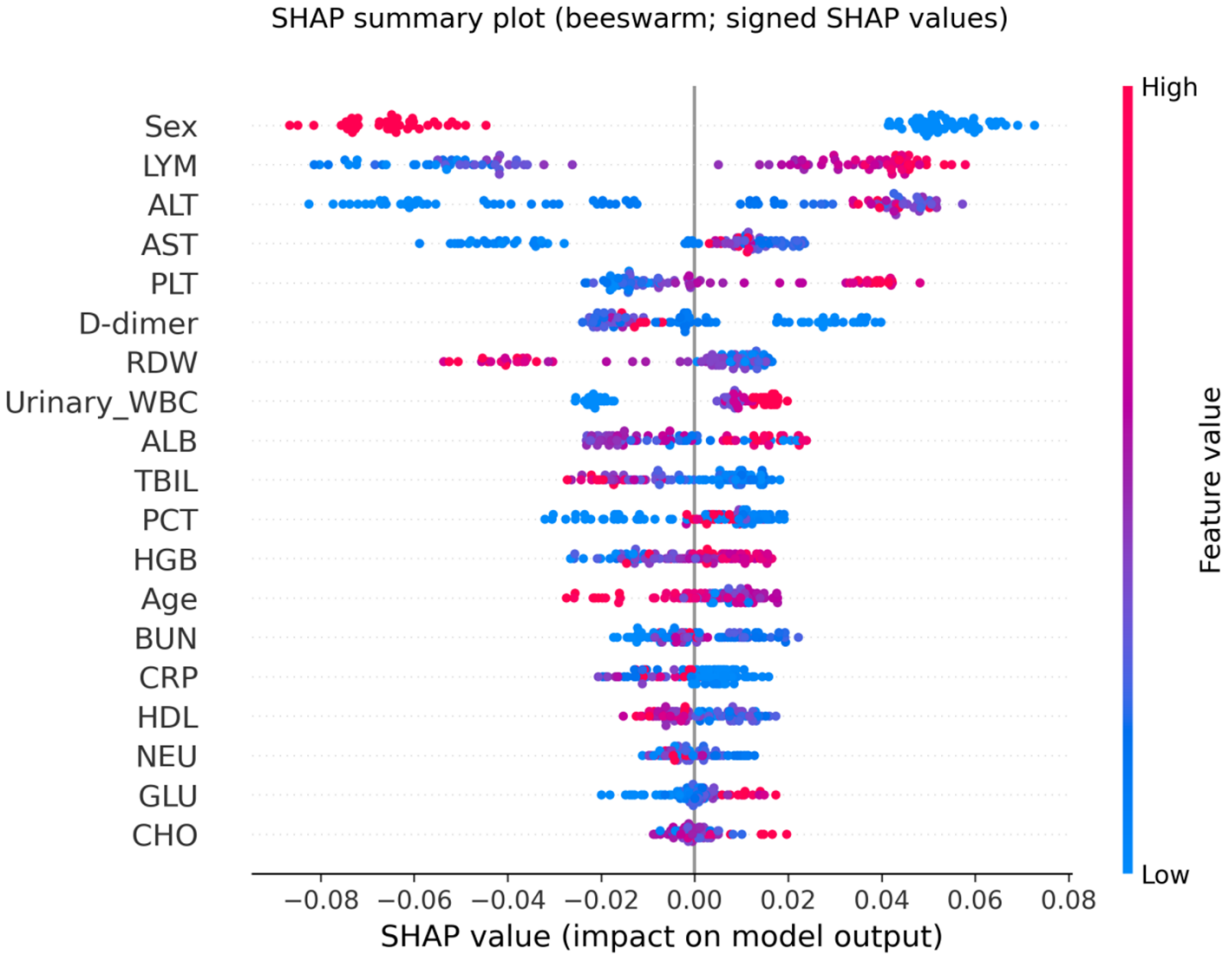
SHAP summary (beeswarm) plot of the Random Forest model. Each dot represents one patient on the held-out test set (n = 93). The x-axis shows signed SHAP values (impact on the prediction of Group=1 (*E. coli*); values > 0 indicate an increased contribution toward predicting *E. coli*, whereas values < 0 indicate a decreased contribution. Dot color encodes the feature value (red = high, blue = low). For Sex (0 = female, 1 = male), red indicates male and blue indicates female.

The SHAP beeswarm plot further illustrated both directionality and inter-individual heterogeneity (**Figure 9**). Positive SHAP values indicate an increased predicted probability of *E. coli* (Group = 1), whereas negative values indicate a decreased probability. For Sex (0 = female, 1 = male), higher values (male) were predominantly associated with negative SHAP values, while lower values (female) were more often associated with positive SHAP values, suggesting that female sex increased and male sex decreased the model-predicted probability of *E. coli* in this cohort. Wider horizontal dispersion for certain variables indicates greater heterogeneity in effects and possible interaction patterns at the patient level.

## Discussion

This study presents a machine learning–based approach for the early identification of *E. coli* in patients with UTIs, leveraging routine clinical and laboratory parameters. The RF classifier achieved moderate diagnostic performance, with an AUC of 0.66 and an average precision of 0.66, demonstrating its feasibility as a clinical decision-support tool. Our microbial profiling confirmed *E. coli* as the predominant uropathogen, accounting for over half of all isolates (51.3%), consistent with global epidemiological trends. *Enterococcus* spp. and *Klebsiella* spp. followed as the next most common agents. These findings align with previous reports underscoring the dominance of *E. coli* in both community and nosocomial UTIs (Hossain et al., 2024; Zhan et al., 2024).

Consistent with existing literature, *E. coli* infections were significantly more common in women, likely due to anatomical predisposition (Johny et al., 2025). Our analysis revealed that *E. coli* infections were associated with significantly elevated absolute lymphocyte counts—a finding that has received limited attention in prior literature. Most existing studies have focused on total leukocyte counts or composite indices such as the neutrophil-to-lymphocyte ratio (NLR) when characterizing the immune response in UTIs (Saheb Sharif-Askari et al., 2020). In contrast, the observed lymphocytosis in *E. coli* UTIs may reflect a distinct immunological signature, potentially linked to adaptive immune activation or pathogen-specific host responses (Hou et al., 2025). To our knowledge, few clinical studies have systematically quantified LYM elevation in *E. coli* UTIs, underscoring the novelty and potential diagnostic relevance of this finding within our cohort, while requiring confirmation in external datasets. Future investigations are warranted to validate this association in larger, multicenter cohorts and to assess whether this signal remains consistent across alternative modeling approaches, to elucidate its mechanistic basis.

Besides LYM elevation, we also observed modest increased serum ALT and ALB levels in patients with *E. coli* UTIs. A Korean pediatric study reported that some UTIs exhibited elevated liver enzyme levels, which normalized following infection resolution, indicating transient hepatic involvement associated with UTIs (Lee et al., 2021). More broadly, systemic infections—including UTIs—can lead to mild to moderate aminotransferase elevations through immune-mediated hepatic stress. Meanwhile, higher ALB levels have been associated with improved outcomes in infection contexts, reflecting better nutritional or immune status, as demonstrated in studies of febrile infections and postoperative UTIs (Wang et al., 2024). Collectively, these findings suggest that slight ALT elevation and ALB may serve as biomarkers of systemic response rather than direct hepatic injury, highlighting their potential utility in distinguishing *E. coli*-driven UTI phenotypes.

*E. coli*–associated UTIs in our cohort were characterized by lower RDW and reduced D-dimer levels compared to non-*E. coli* infections, suggesting pathogen-specific hematologic and coagulative responses. While elevated RDW and D-dimer are well-documented markers of severe infections—particularly Gram-negative bacteremia and sepsis—they are more typically associated with greater illness severity and poor prognosis, rather than pathogen-specific differences (Xia et al., 2021). For example, higher RDW has been linked with adverse outcomes in sepsis, whereas elevated D-dimer reflects systemic inflammation and thrombotic activity, and its increase is nonspecific across bacterial infections (Lee et al., 2018). The comparatively lower levels of these markers in *E. coli* UTIs may indicate a milder systemic response or less extensive endothelial activation relative to other pathogens. Although these findings require further investigation, they point towards potentially valuable clues for early pathogen differentiation and deserve exploration in larger prospective cohorts.

Interpretability was primarily based on SHAP (TreeSHAP), which provides signed, patient-level attributions and can capture nonlinear and interaction effects (Rodríguez-Pérez and Bajorath, 2020). To contextualize the RF model and reduce the risk of model-specific findings, we benchmarked RF against commonly used baseline classifiers, including regularized logistic regression, SVM, and gradient boosting. This benchmarking reduces the risk of model-specific artifacts and allows us to more robustly interpret the features identified as significant. Because routinely collected laboratory variables can be correlated, feature attributions may be shared across correlated predictors; therefore, the top SHAP contributors should be interpreted as candidate predictors rather than definitive pathogen-specific biomarkers. In addition, SHAP provides local explanations for individual predictions, which can support clinical review and improve transparency and trust in model outputs (Janssen et al., 2022; Bifarin, 2023).

The RF model achieved moderate discriminative performance (AUC = 0.66), which was confirmed in our benchmarking comparisons. Nevertheless, it offers clinical value due to the rapid and noninvasive nature of the input features. The model also demonstrated balanced precision and recall between *E. coli* and non-*E. coli* UTIs, helping to minimize classification bias. Accordingly, it may serve as a supportive triage tool to prioritize early decision-making while awaiting culture-based confirmation.

### Limitations and future directions

This study is limited by its single-center, retrospective design, and external validation is needed to confirm generalizability across diverse patient populations. Although multiple models (Random Forest, logistic regression, SVM, and gradient boosting) were compared, the findings should be interpreted within the context of the dataset and applied classifiers. Future research should assess the stability of biomarkers across different algorithms and evaluate model performance in external cohorts. Additionally, prospective validation and temporal testing are needed to assess the model’s generalizability and clinical applicability over time.

## Conclusions

In this study, we developed an explainable machine learning model to facilitate the early identification of *E. coli* urinary tract infections using routine clinical data. The random forest classifier demonstrated moderate discriminative performance (AUC = 0.66), with balanced precision and recall across pathogen classes, underscoring its potential clinical applicability. SHAP-based interpretability revealed important, and in some cases non-linear, feature interactions—most notably the strong predictive role of sex and LYM—offering a transparent framework for AI (artificial intelligence)-driven decision support.

Notably, modest elevations in ALT and ALB levels, alongside lower RDW and D-dimer in *E. coli* infections, may represent distinct systemic responses, meriting further investigation. These findings collectively highlight the feasibility of integrating interpretable machine learning with conventional biomarkers to enhance diagnostic efficiency, reduce reliance on empirical therapy, and inform targeted interventions in urinary tract infection management.

## Data Availability

The raw data supporting the conclusions of this article will be made available by the authors, without undue reservation.
