# Supplementary figures and images for: Explainable machine learning for early detection of Escherichia coli urinary tract infections: integrating SHAP interpretation and bacterial epidemiology
Source: Front Cell Infect Microbiol. 2026 Feb 13;16:1740707. doi: 10.3389/fcimb.2026.1740707 (PMC12946121; doi:10.3389/fcimb.2026.1740707)

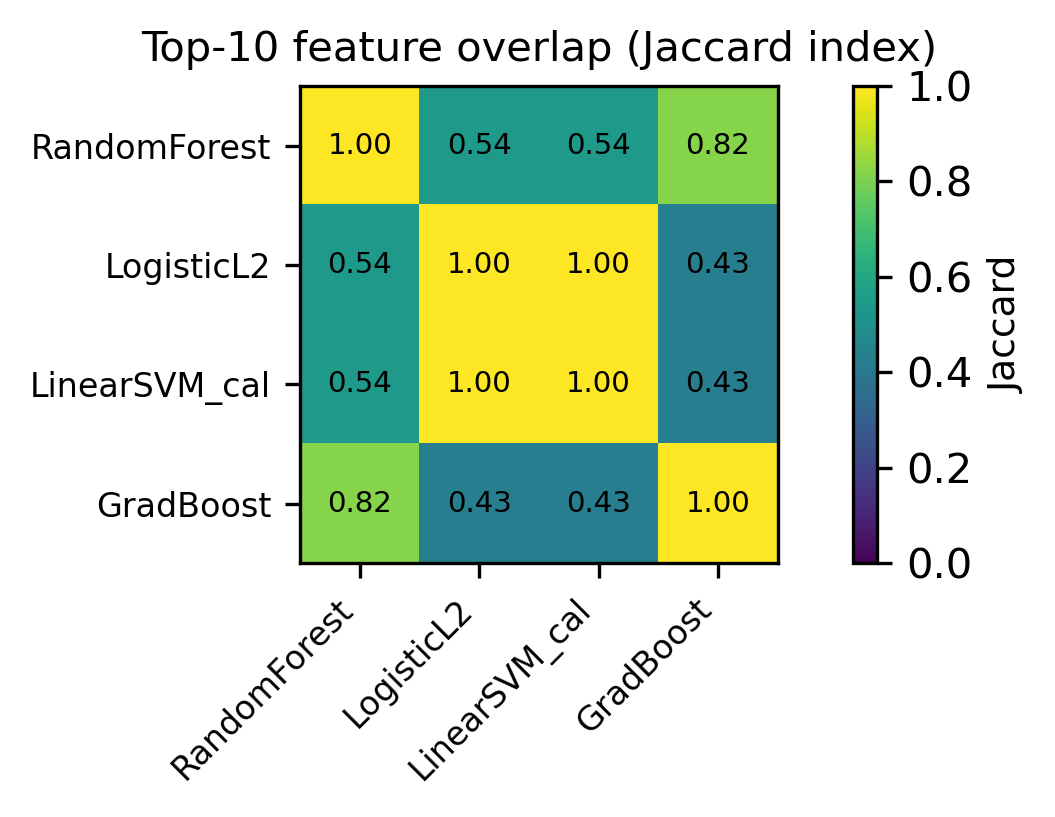

Supplement: Supplementary file 1 [file Image1.png]

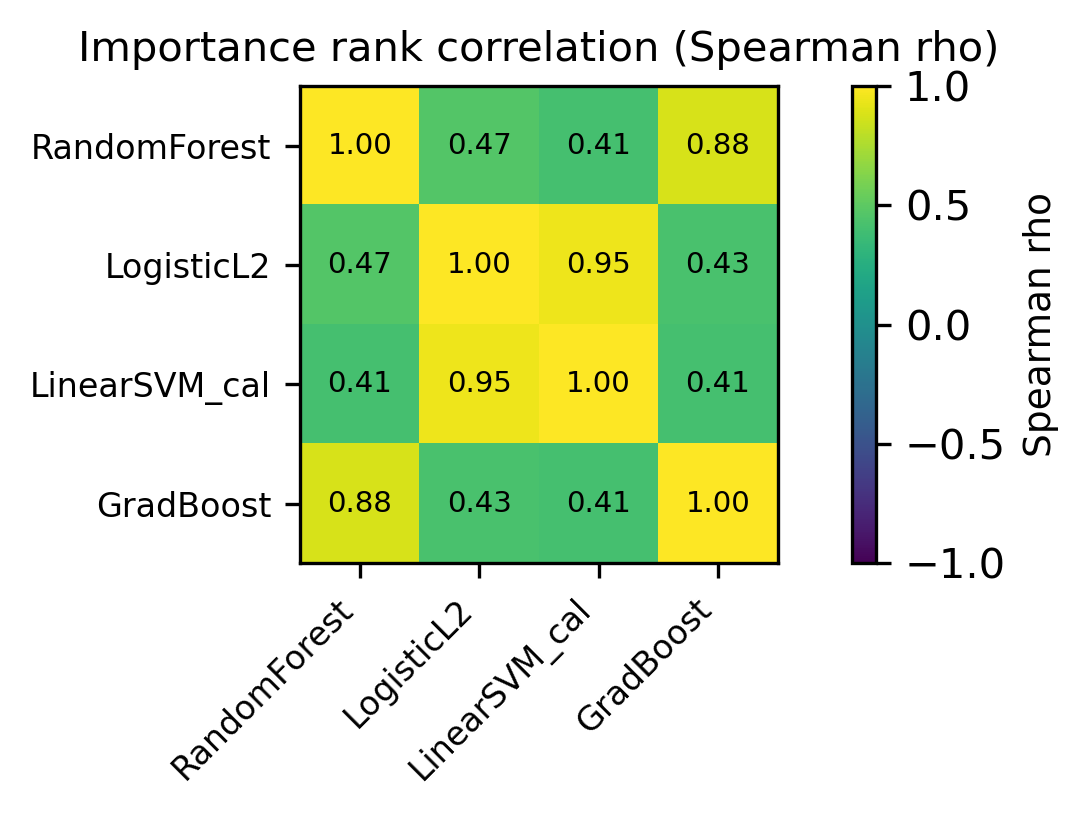

Supplement: Supplementary file 2 [file Image2.png]
