# Supplementary material for: Explainable machine learning for early detection of Escherichia coli urinary tract infections: integrating SHAP interpretation and bacterial epidemiology
Source: Front Cell Infect Microbiol. 2026 Feb 13;16:1740707. doi: 10.3389/fcimb.2026.1740707 (PMC12946121; doi:10.3389/fcimb.2026.1740707)
Supplement: Supplementary file 3 [file Table1.docx]

**Supplementary Table S2. Missingness rates of predictors and final feature dimensionality**

| Variable | Type | Encoding (final input) | Missing, n | Missing, % | Imputation in pipeline |
| --- | --- | --- | --- | --- | --- |
| Sex | Binary | Binary-coded (0/1) | 1 | 0.32 | Median (training-set only) |
| Urinary_WBC | Ordinal | Ordinal-coded (ordered grades) | 3 | 0.97 | Median (training-set only) |
| Age | Continuous | Numeric (continuous) | 0 | 0 | Median (training-set only) |
| HGB | Continuous | Numeric (continuous) | 0 | 0 | Median (training-set only) |
| RDW | Continuous | Numeric (continuous) | 5 | 1.62 | Median (training-set only) |
| LYM | Continuous | Numeric (continuous) | 0 | 0 | Median (training-set only) |
| NEU | Continuous | Numeric (continuous) | 0 | 0 | Median (training-set only) |
| PLT | Continuous | Numeric (continuous) | 0 | 0 | Median (training-set only) |
| CRP | Continuous | Numeric (continuous) | 0 | 0 | Median (training-set only) |
| PCT | Continuous | Numeric (continuous) | 10 | 3.25 | Median (training-set only) |
| ALT | Continuous | Numeric (continuous) | 0 | 0 | Median (training-set only) |
| AST | Continuous | Numeric (continuous) | 0 | 0 | Median (training-set only) |
| GLU | Continuous | Numeric (continuous) | 0 | 0 | Median (training-set only) |
| ALB | Continuous | Numeric (continuous) | 7 | 2.27 | Median (training-set only) |
| BUN | Continuous | Numeric (continuous) | 0 | 0 | Median (training-set only) |
| TBIL | Continuous | Numeric (continuous) | 0 | 0 | Median (training-set only) |
| CHO | Continuous | Numeric (continuous) | 0 | 0 | Median (training-set only) |
| HDL | Continuous | Numeric (continuous) | 0 | 0 | Median (training-set only) |
| D-dimer | Continuous | Numeric (continuous) | 0 | 0 | Median (training-set only) |

Analytic cohort: n = 308. Predictors: p = 19. Sex was binary-coded and urinary leukocyte grade (Urinary_WBC) was ordinal-coded; no one-hot expansion was applied. Missing values (if present) were imputed using the median estimated from the training set within the modeling pipeline.
