# Supplementary material for: Explainable machine learning for early detection of Escherichia coli urinary tract infections: integrating SHAP interpretation and bacterial epidemiology
Source: Front Cell Infect Microbiol. 2026 Feb 13;16:1740707. doi: 10.3389/fcimb.2026.1740707 (PMC12946121; doi:10.3389/fcimb.2026.1740707)
Supplement: Supplementary file 4 [file Table2.docx]

**Supplementary Table S2.** Classification performance of the random forest model on the test set (n=93)

| Class | Precision | Recall | F1-score | Support |
| --- | --- | --- | --- | --- |
| Non-*E. coli* group | 0.61 | 0.67 | 0.64 | 45 |
| *E. coli* group | 0.66 | 0.60 | 0.63 | 48 |
| Accuracy |  |  | 0.63 | 93 |
| Macro avg | 0.64 | 0.64 | 0.63 | 93 |
| Weighted avg | 0.64 | 0.63 | 0.63 | 93 |

Note: Macro average reflects the unweighted mean of metrics across both classes, while weighted average accounts for the relative class sizes (support). The model demonstrated balanced performance in distinguishing *E. coli* and non-*E. coli* UTI, with no significant class imbalance in predictive capability.
